# A Closer Look: Familial Adenomatous Polyposis Suspected Through Ophthalmological Findings in an Adolescent

**DOI:** 10.7759/cureus.82857

**Published:** 2025-04-23

**Authors:** João D Freitas, Miguel R Ferreira, Daniela Mota, Sara Marante, Sandra Matapa

**Affiliations:** 1 Family Medicine, Unidade de Saúde Familiar de Baião - Unidade Local de Saúde do Tâmega e Sousa, Porto, PRT; 2 Family Medicine, Unidade de Saúde Familiar de Resende - Unidade Local de Saúde do Tâmega e Sousa, Porto, PRT

**Keywords:** adenomatous polyposis coli, colectomy, colonoscopy, fundus oculi, genetic testing, visual acuity

## Abstract

Familial adenomatous polyposis (FAP) is a hereditary condition characterized by the early onset of hundreds to thousands of adenomatous colorectal polyps, with a high risk of colorectal cancer if untreated. While genetic testing and gastrointestinal symptoms often prompt diagnosis, certain extraintestinal manifestations, such as congenital hypertrophy of the retinal pigment epithelium (CHRPE), may offer early diagnostic clues. This case describes a female adolescent whose initial complaint was decreased visual acuity. This prompted an examination that revealed bilateral pigmented retinal lesions consistent with CHRPE, which subsequently led to the suspicion of FAP, despite the absence of gastrointestinal complaints or known familial mutations. Subsequent genetic testing confirmed a pathogenic variant in the APC gene, and colonoscopy revealed extensive polyposis. This case highlights the importance of recognizing ophthalmological manifestations as potential early indicators of inherited colorectal cancer syndromes. It also underscores the relevance of a multidisciplinary approach in managing complex hereditary diseases, with respect for patient autonomy and shared decision-making.

## Introduction

Familial adenomatous polyposis (FAP) is an autosomal dominant hereditary disorder, accounting for approximately 1% of all colorectal cancers [1]. It is caused by pathogenic variants in the APC tumor suppressor gene, located on chromosome 5q21-q22, with over 1000 distinct mutations described in families presenting both classic and attenuated forms of FAP [2,3].

The classic form is typically characterized by the development of more than 100 up to thousands of adenomatous polyps throughout the colon and rectum. These polyps usually begin to appear in early adolescence and, if untreated, carry a nearly 100% lifetime risk of progressing to colorectal carcinoma [4]. Clinically, gastrointestinal symptoms such as abdominal pain, diarrhea, or lower gastrointestinal bleeding may be present [5], although the majority of carriers remain asymptomatic in the initial stages.

In addition to colorectal findings, FAP may present with extracolonic manifestations, which can vary in severity and distribution depending on the underlying genetic mutation [6]. These may include duodenal and ampullary adenomas [7], desmoid tumors [8], papillary thyroid carcinoma [9], and ocular lesions such as congenital hypertrophy of the retinal pigment epithelium (CHRPE) [10].

The diagnosis of FAP is primarily based on colonoscopic findings, supported by family history, and definitively confirmed through genetic testing [11]. Management involves prophylactic colectomy, with surgical options including total proctocolectomy with terminal ileostomy, total proctocolectomy with ileal pouch-anal anastomosis, or total colectomy with ileorectal anastomosis [12]. Long-term surveillance strategies must also address the risk of extracolonic neoplasms.

Despite the genetic and clinical complexity of FAP, early diagnosis remains a challenge, particularly when initial manifestations are atypical or extraintestinal. In rare cases, ophthalmologic findings such as CHRPE may precede gastrointestinal symptoms and raise suspicion of polyposis syndromes [13].

This case report describes an adolescent patient in whom decreased visual acuity prompted ophthalmologic evaluation, ultimately leading to the diagnosis of FAP. The case underscores the value of multidisciplinary collaboration and highlights the diagnostic significance of ophthalmologic signs such as CHRPE in hereditary colorectal cancer syndromes.

## Case presentation

**Figure 1:**
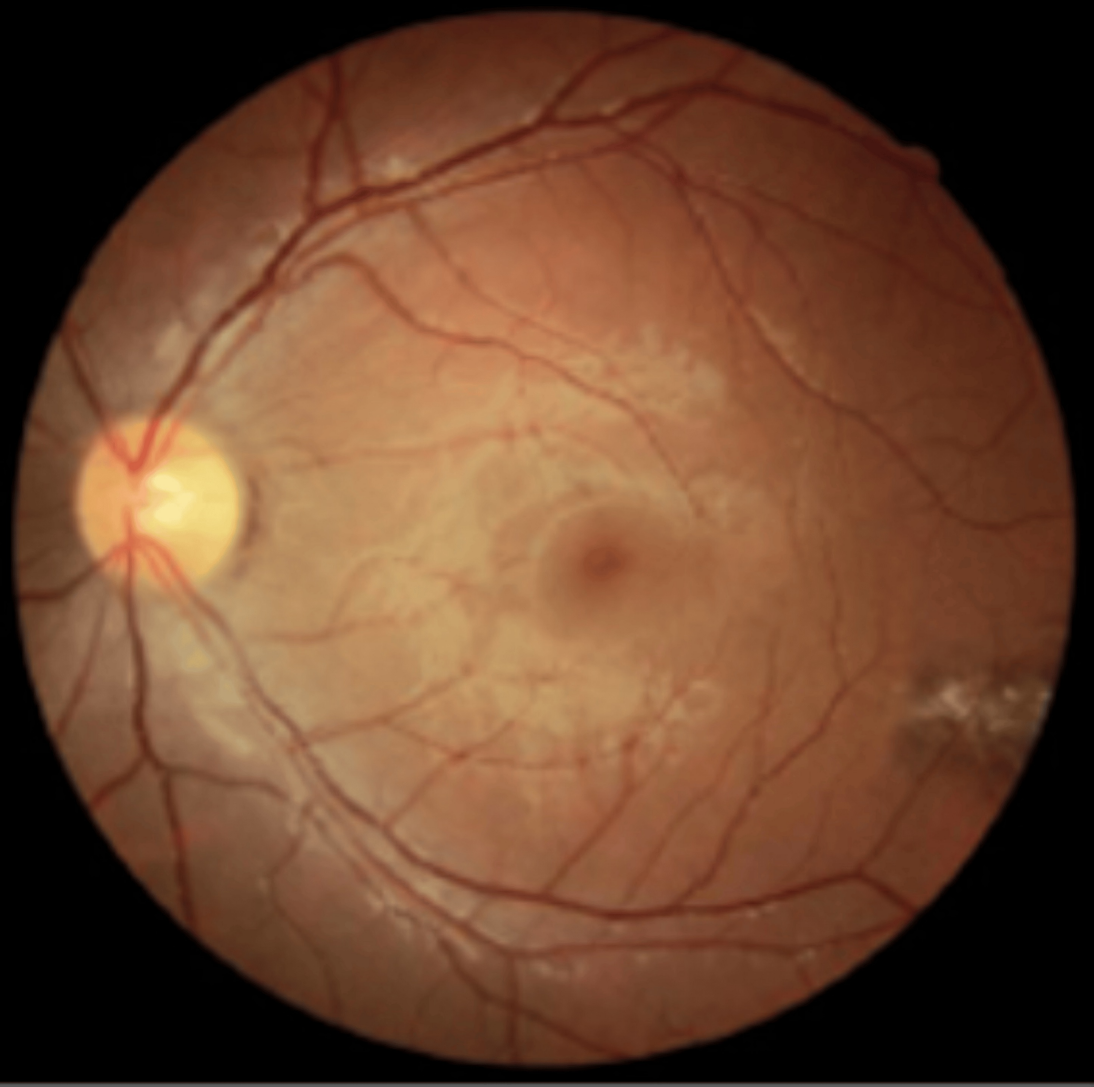
Ocular fundus

**Figure 2:**
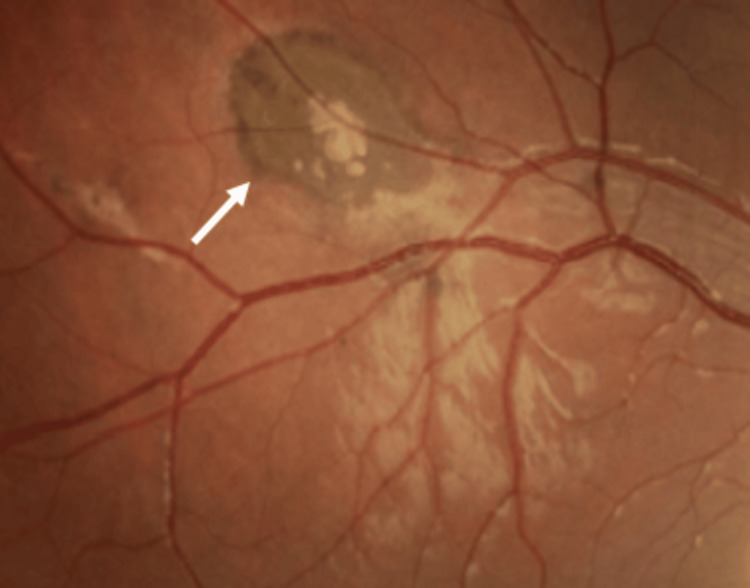
Ocular fundus (right side): pigmented lesions with a comet-tail atrophy halo

**Figure 3:**
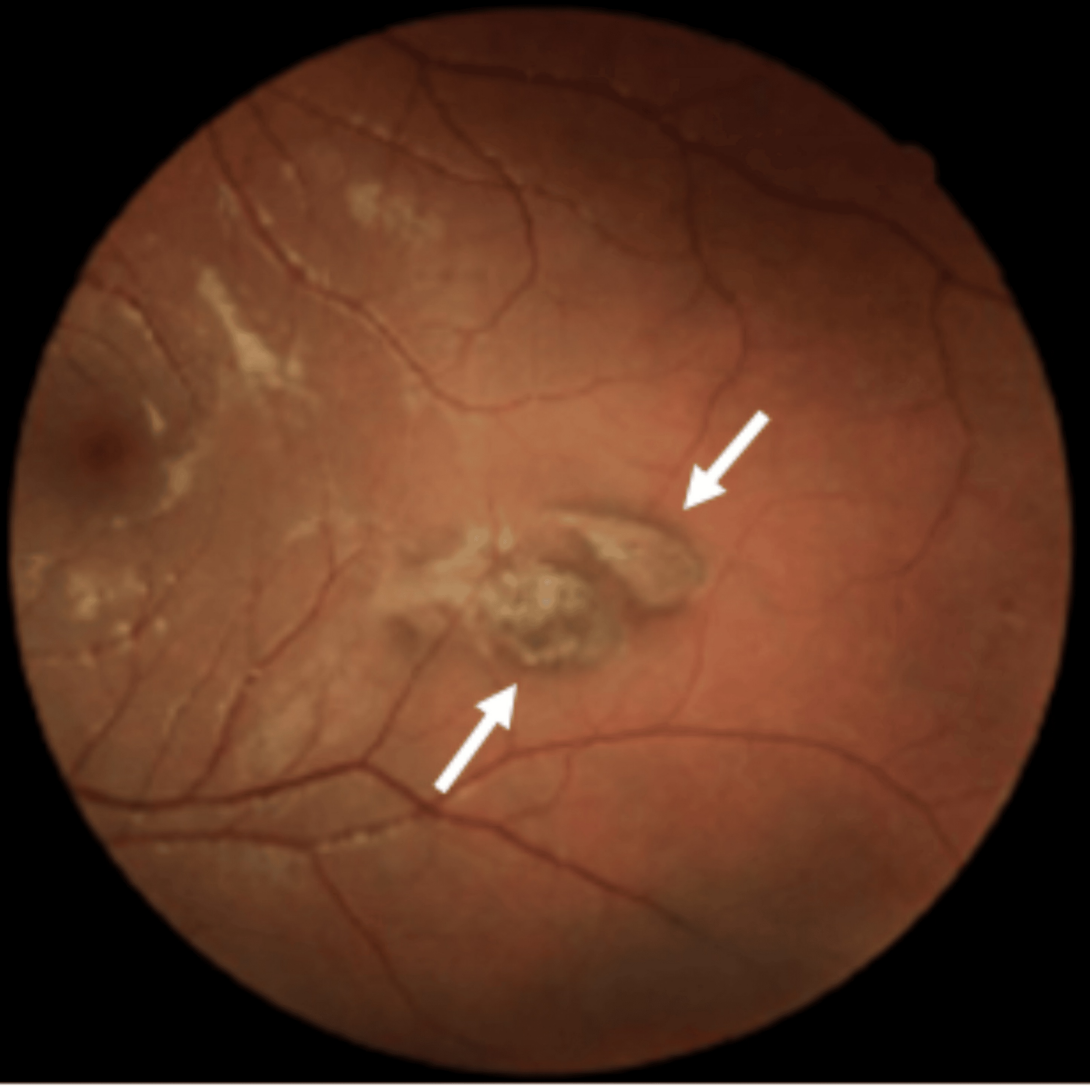
Ocular fundus (left side): pigmented lesions with a comet-tail atrophy halo

A female adolescent with a history of allergic rhinitis and atopic dermatitis and no regular medication presented to our Family Medicine unit at the age of 14, following a progressive decrease in visual acuity. She had previously consulted an ophthalmologist, who identified multiple bilateral pigmented retinal “comet-tailed” lesions suggestive of CHRPE (Figures 1, 2, 3). Based on these findings, the ophthalmologist issued a referral letter recommending a gastrointestinal evaluation, which the patient delivered to her primary care physician.

**Figure 4:**
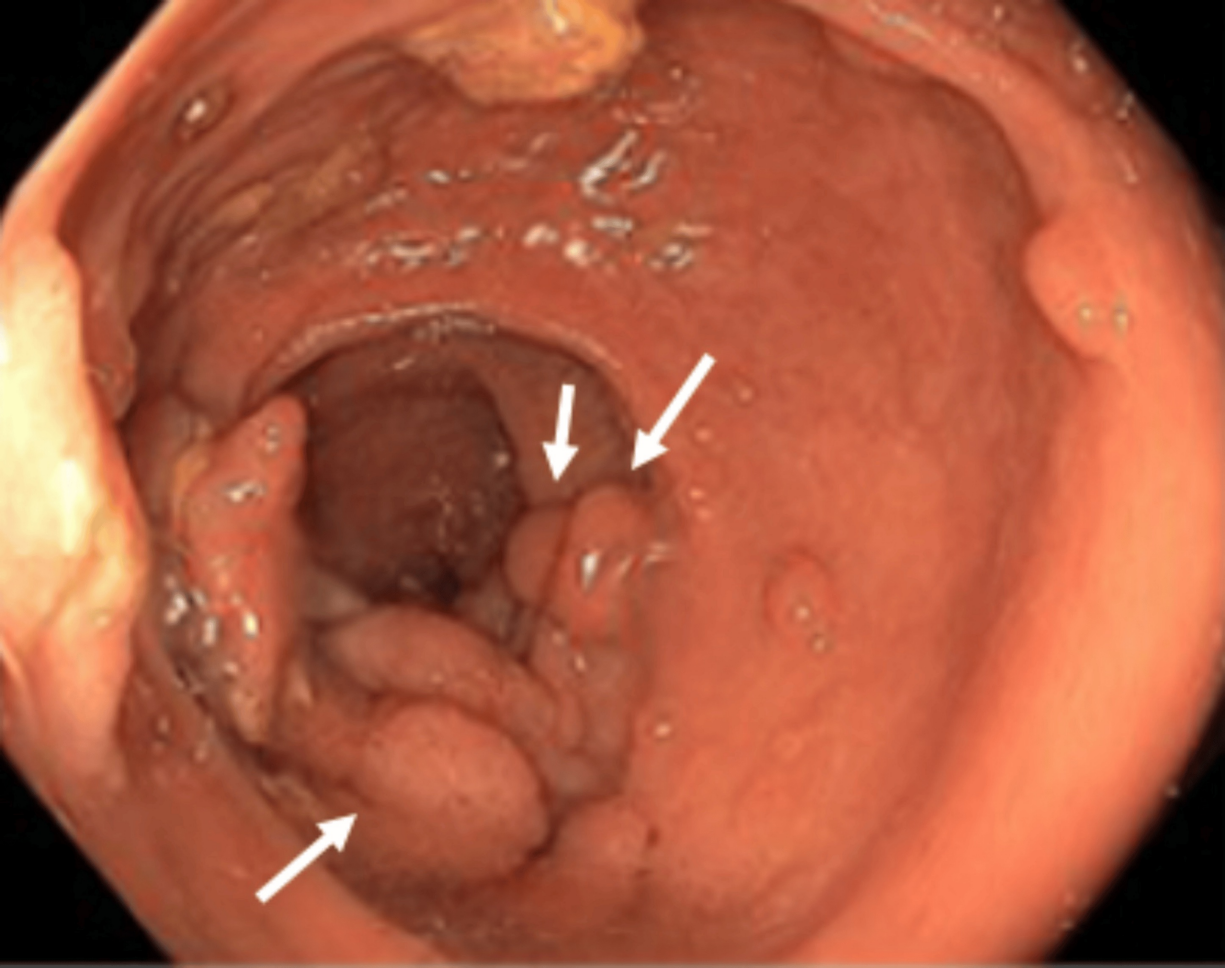
Rectum: numerous adenomatous polyps

**Figure 5:**
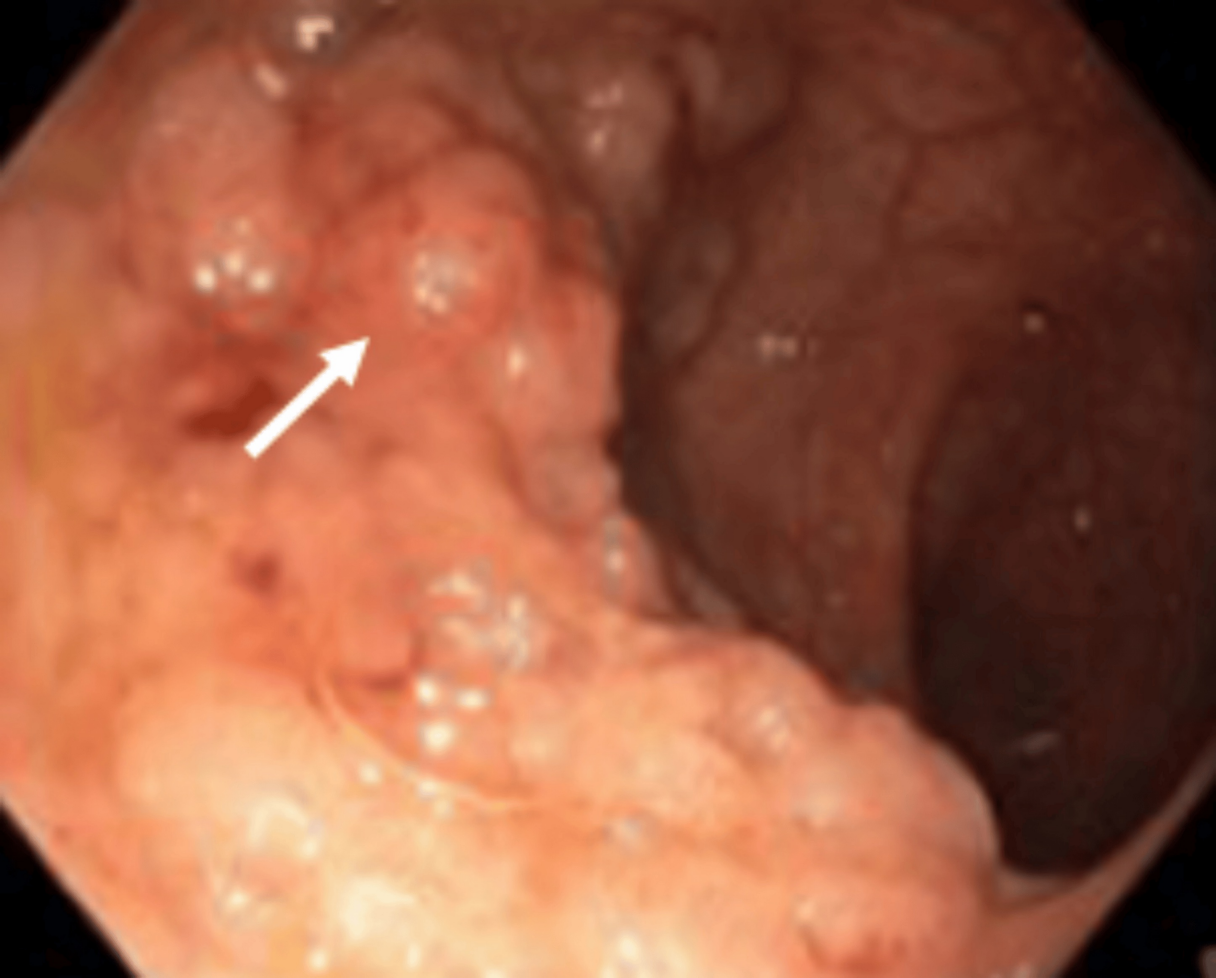
Colon: numerous adenomatous polyps

At the time, although she was asymptomatic from a gastrointestinal standpoint and no relevant family history was reported, the ophthalmologic findings raised strong clinical suspicion for a hereditary polyposis syndrome. She was referred to the Gastroenterology department, where genetic testing was requested and a colonoscopy was scheduled. The genetic test confirmed a pathogenic mutation in the APC gene (c.3183_3187delACAAA p.(Gln1062*)), establishing the diagnosis of FAP. A colonoscopy performed shortly thereafter revealed between 500 and 1000 adenomatous polyps distributed throughout the colon, some measuring up to 25 mm, including flat lesions predominantly located in the distal colon and rectum, without evidence of submucosal invasion (Figures 4, 5). Histopathological examination of three colonic mucosal samples showed tubulovillous adenomatous transformation with low-grade dysplasia. In light of these findings, a structured surveillance program was initiated.

The patient underwent close follow-up by a multidisciplinary team including gastroenterology, ophthalmology, genetics, and general surgery. During this period, she was monitored for both colorectal and extracolonic manifestations of FAP. As part of the preoperative assessment, routine laboratory workup revealed microcytic hypochromic anemia (hemoglobin: 10.7 g/dL; MCV: 71.1 fL; MCH:22; ferritin: 9 ng/mL; transferrin saturation: 3%), consistent with iron deficiency anemia (Table 1). This was attributed to chronic occult gastrointestinal blood loss secondary to extensive colorectal polyposis. Supplementation with oral iron was initiated with partial improvement. 

**Table 1:** Hematological and iron profile of the patient This table presents the results of the complete blood count and iron studies obtained in a female teenager diagnosed with familial adenomatous polyposis (FAP). The findings are consistent with microcytic, hypochromic anemia due to iron deficiency, characterized by low hemoglobin, hematocrit, mean corpuscular volume (MCV), mean corpuscular hemoglobin (MCH), serum iron, ferritin, and transferrin saturation, along with elevated red cell distribution width (RDW).

Parameter	Result	Reference range
Red blood cells (RBCs)	4.85 x10⁶/μL	4.1-5.1
Hemoglobin	10.7 g/dL	12-16
Hematocrit	34.50%	36.0-46.0
MCV - mean corpuscular volume	71.1 fL	78-102
MCH - mean corpuscular hemoglobin	22.0 pg	25.0-35.0
MCHC - mean corpuscular hemoglobin conc.	30.9 g/dL	31.0-37.0
RDW - red cell distribution width	16.70%	11.5-14.5
HDW - hemoglobin distribution width	2.83 g/dL	2.20-3.20
Platelets	291 x10³/μL	150-400
MPV - mean platelet volume	8.0 fL	7.2-11.1
PDW - platelet distribution width	45.40%	25.0-65.0
PCT - plateletcrit	0.23%	0.12-0.36
White blood cells (WBC)	7.40 x10³/μL	4.50-13.00
Neutrophils (%)	60.10%	40.0-75.0
Lymphocytes (%)	22.20%	20.0-45.0
Monocytes (%)	11.70%	2.0 -10.0
Eosinophils (%)	2.90%	1.0-6.0
Basophils (%)	0.40%	0.0-1.0
Large mononuclear cells (%)	2.70%	0.0-4.0
Neutrophils (absolute)	4.5 x10³/μL	1.80-8.00
Lymphocytes (absolute)	1.6 x10³/μL	1.20-5.20
Monocytes (absolute)	0.9 x10³/μL	0.40-0.50
Eosinophils (absolute)	0.2 x10³/μL	0.20-0.30
Basophils (absolute)	0.0 x10³/μL	0.02-0.10
Large mononuclear cells (absolute)	0.2 x10³/μL	0.00-0.44
Iron	15 μg/dL	50-150
Transferrin	341 mg/dL	200-370
Transferrin saturation	3%	15-45
Ferritin	9 ng/mL	2.20-178
Vitamin B12	379 pg/mL	191-663
Folic Acid (Folate)	4.4 ng/mL	3.9 – 26.8

To reduce adenoma burden and potentially delay the need for colectomy, the patient was prescribed celecoxib (a selective COX-2 inhibitor), in line with established chemopreventive strategies in FAP.

As part of endoscopic surveillance, at the age of 17 years old, she underwent excision of a flat rectal lesion, which revealed a tubulovillous adenoma with low-grade dysplasia. Several months later, seven additional rectal flat polyps were removed; histology confirmed tubulovillous adenomas with areas of both low- and high-grade dysplasia.

In addition, complementary imaging studies, including thyroid ultrasound and upper GI endoscopy, were performed. No abnormalities were detected, and no evidence of desmoid tumors, duodenal or ampullary adenomas, thyroid cancer, or central nervous system lesions was found.

The diagnosis of FAP and the potential functional repercussions associated with the proposed surgery had a significant emotional and psychological impact on the patient and her family, which led them to postpone definitive treatment. At the age of 18, after completing high school and preparing for a new stage in her life, she agreed to proceed with surgery on the condition that rectal preservation would be pursued. A total colectomy with ileorectal anastomosis was successfully performed via robotic surgery. The procedure was well tolerated, with no perioperative complications

The patient remains under regular follow-up for monitoring of the remaining rectum and surveillance of extracolonic manifestations. Endoscopic and genetic screening of first-degree relatives was negative.

## Discussion

This is a sporadic case of classic FAP, with an unusual initial suspicion raised by ophthalmology. FAP follows an autosomal dominant inheritance pattern, with nearly complete penetrance for colorectal polyposis and variable penetrance for extracolonic manifestations. Up to 25% of FAP cases result from de novo APC mutations, meaning these patients do not have a family history of FAP but remain at risk of transmitting the disease to their offspring [14]. In this case, genetic testing confirmed the presence of a pathogenic variant in the APC gene (c.3183_3187delACAAA p.(Gln1062*)), consistent with a diagnosis of FAP. This variant results in a premature stop codon and subsequent production of a truncated, non-functional APC protein. The pathogenesis of FAP is rooted in mutations in the APC tumor suppressor gene, located on chromosome 5q21-q22. According to the “two-hit hypothesis,” a second somatic mutation leads to biallelic inactivation, loss of heterozygosity, and tumorigenesis. This mechanism promotes adenoma formation and accounts for both colorectal and extracolonic manifestations. Early genetic diagnosis enables prophylactic measures, personalized surveillance, and cascade testing of at-risk relatives, which are crucial for preventing malignant transformation and improving prognosis [13].

Although this patient presented with CHRPE, which is a known extracolonic manifestation associated with both classic FAP and Gardner’s syndrome, no other characteristic features of Gardner’s syndrome, such as osteomas, epidermoid cysts, or dental anomalies, were observed. Similarly, no central nervous system tumors, particularly medulloblastomas, were detected, which are hallmark features of Turcot syndrome. Current evidence supports the view that both Gardner’s and Turcot syndromes represent phenotypic variants of FAP, rather than distinct clinical entities, all stemming from germline mutations in the APC gene. Therefore, based on the confirmed APC pathogenic variant and the absence of additional specific manifestations, the diagnosis of classic FAP was considered more appropriate. Nevertheless, ongoing surveillance remains crucial, as extracolonic features may still emerge over time and warrant re-evaluation of the clinical subtype [13].

Multidisciplinary follow-up persists imperative for surveillance of rectal neoplasia. She continues under periodic follow-up in Surgery, Gastroenterology, and Ophthalmology. Upper gastrointestinal endoscopy is recommended every four to five years starting at 20-25 years old due to the association of FAP with duodenal adenomas and gastric polyps/adenocarcinoma, with duodenal adenomas present in over 50% of cases in some studies [12,13,15]. Ophthalmologic follow-up every 12-18 months is warranted to monitor potential progression of CHRPE lesions, which may occur in 46-83% of cases and, in some instances, affect visual acuity or be associated with pigmented nodular adenocarcinomas [16-18]. In addition, thyroid ultrasound surveillance every two to five years is recommended from late adolescence, given the 12% prevalence of thyroid carcinoma in FAP patients [19].

Anemia, particularly iron deficiency anemia, is a common issue in patients with FAP, primarily due to chronic gastrointestinal bleeding from the polyps. In this case, the patient exhibited low hemoglobin levels (10.7 g/dL) and iron deficiency (ferritin 9 ng/mL, iron 15 μg/dL, transferrin saturation 3%), indicative of iron deficiency anemia. This type of anemia can be complicated further by the chronic inflammatory state often present in hereditary colorectal cancer syndromes, which may contribute to impaired iron absorption and utilization. Addressing anemia in FAP patients is essential to improve their overall well-being and ensure they are fit for surgical interventions such as colectomy. Early identification and management of anemia can also help mitigate risks during surgical procedures and postoperative recovery [13]. In addition, the use of celecoxib has been explored as a potential adjunctive therapy in FAP management due to its role in inhibiting COX-2, an enzyme involved in polyp formation and progression. Celecoxib has been shown to reduce the number of adenomas in some patients with FAP, although it is not a replacement for prophylactic colectomy. Long-term use and the effectiveness of celecoxib are still subjects of ongoing research. In this case, celecoxib was considered to help prevent further adenomatous growth, in conjunction with regular surveillance and monitoring [13].

Early ophthalmologic suspicion of CHRPE - a known extracolonic manifestation of FAP - triggered further evaluation in primary care, leading to a referral for genetic testing and gastrointestinal investigation. This cascade of coordinated actions across multiple levels of care enabled diagnosis before the onset of gastrointestinal symptoms and the development of colorectal cancer. If this patient had not been managed in a collaborative, multidisciplinary manner, the diagnosis of FAP could have been delayed - a situation associated with a significantly increased risk of colorectal cancer. Evidence supports that coordinated care through specialized services and registries improves diagnosis, surveillance, and long-term outcomes in patients with FAP. Early diagnosis and proper surveillance can significantly reduce mortality, emphasizing the critical role of genetic counseling, endoscopy, and surgery services for long-term management [13].

This case highlights the central role of patient autonomy in clinical decision-making, particularly in the context of chronic hereditary diseases such as FAP. Despite the recommendation for early prophylactic colectomy following diagnosis at age 15, the patient and her family expressed significant emotional and psychological distress regarding the implications of surgery. These concerns led them to postpone the intervention until she completed secondary education and felt prepared for the physical and lifestyle changes involved. This shared decision-making process demonstrates the importance of aligning medical recommendations with patient values and life context - a principle supported by modern medical ethics and international guidelines on patient-centered care and shared decision-making. Respecting patient autonomy is associated with improved satisfaction, adherence to care, and long-term outcomes. Ultimately, the patient underwent surgery at age 18 under conditions that preserved quality of life and respected her personal goals, illustrating the effectiveness of a patient-centered approach that integrates evidence-based practice with individualized care [20].

## Conclusions

The early ophthalmologic suspicion of FAP in this patient, without a relevant family history, illustrates how extraintestinal manifestations can be pivotal in identifying hereditary colorectal cancer syndromes. Despite some delays in diagnosis and treatment acceptance, coordination between primary and secondary care ultimately enabled appropriate surveillance and surgical management.

This case also underscores the importance of multidisciplinary vigilance and the need to strengthen integrated care systems to prevent potential mismanagement in complex, hereditary conditions. Further studies are needed to explore how to optimize early detection and coordination across care levels in similar cases.
